# Are powder-technology-built stems safe? A midterm follow-up registry study

**DOI:** 10.1007/s10856-020-06481-8

**Published:** 2021-01-20

**Authors:** Francesco Pardo, Barbara Bordini, Francesco Castagnini, Federico Giardina, Cesare Faldini, Francesco Traina

**Affiliations:** 1 Ortopedia-Traumatologia e Chirurgia Protesica e dei Reimpianti d’anca e di Ginocchio, IRCCS Istituto Ortopedico Rizzoli, Via G.C. Pupilli 1, Bologna, 40136 Italy; 2 Laboratorio di Tecnologia Medica, IRCCS Istituto Ortopedico Rizzoli, Via di Barbiano 1/10, Bologna, 40136 Italy; 3 Clinica Ortopedica e Traumatologica I, IRCCS Istituto Ortopedico Rizzoli, Via G.C. Pupilli 1, Bologna, 40136 Italy; 4 DIBINEM, University of Bologna, Bologna, Italy; 5 University of Messina, Messina, Italy

## Abstract

**Background:**

Powder technology was developed to bring together the mechanical features and high porosity of titanium. However, the high porosity may theoretically compromise mechanical resistance. Literature is deficient about the use and safety profile of cementless femoral implants built using additive manufacturing (in particular electron beam melting technology, EBM). The purpose of this study was to evaluate the survival rates and the reason for revisions (especially implant breakage) of the first two EBM-built stems at a mid-term follow-up, using a joint arthroplasty registry.

**Methods:**

The registry of Prosthetic Orthopedic Implant (RIPO) was investigated about cementless stems implanted from 2010 to 2017. Stems built with EBM technology (Parva and Pulchra stems; Adler Ortho, Milan, Italy) were compared to all the other cementless stems implanted during the same period, acting as control group. The survival rates and reasons for revision were assessed.

**Results:**

No stem breakage occurred. At 5-year follow-up, the survival rates of the two cohorts were not statistically different (96.8% EBM stems, 98.0% standard cementless stems; *p* > 0.05). In the EBM stems, aseptic loosening occurred in 1.7% of the cases at the latest follow-up.

**Conclusions:**

This large cohort showed that mechanical resistance is not a concern in EBM stems at mid-term follow-up. However, larger populations and longer follow-ups are needed to further validate these results.

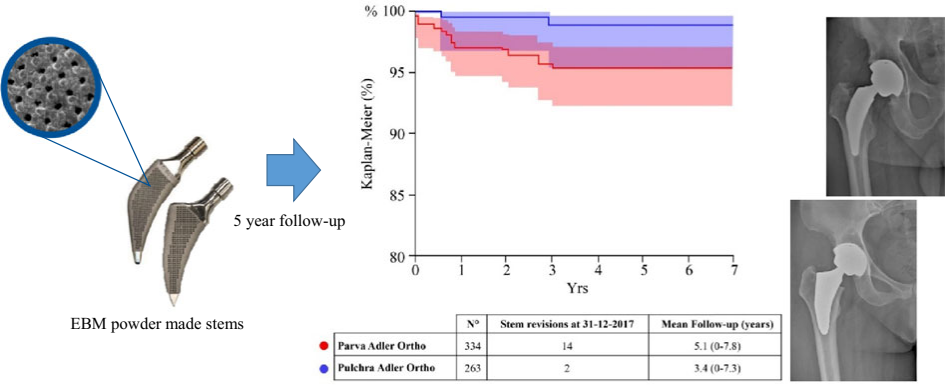

## Introduction

Cementless stems achieved dependable long-term results in total hip arthroplasty (THA) [1]: to date, cementless femoral fixation is generally favored in Europe and North America [2]. Despite the fact that traditional implants showed excellent results, many new implant designs and materials were introduced on the market with the aim of improving bone ingrowth and allowing minimally invasive approaches.

Among cementless femoral stems, Ti6Al4V implants tend to mimic the elastic modulus of bone, outperforming cobalt-chromium alloys [3]. Moreover, Titanium alloy implants showed high resistance to breakage and high biocompatibility, as well as bone-bonding ability [4].

Recently, an innovative method has been developed to manufacture high porosity titanium implants, electron beam melting technology (EBM) [5]. This technique merges the extreme malleability of titanium with the high osseointegrative property of porous surface. This process, performed by an electron beam gun in a vacuum chamber, maintains the high purity and high strength of titanium components with no risk of delamination [5, 6]. This additive manufacturing provides a bulk construct with a constant porosity (65%), each pore size was 700 μm. This pore size was shown to achieve optimal osseointegrative attitude in both in vivo and in vitro studies [7, 8]. EBM technology has already been evaluated in acetabular cups [9, 10]. In a 7-year follow-up registry study, the aseptic loosening rate of highly porous titanium cups in cementless THA was three times lower than all other cementless cups.

The application of EBM technology to the stems aims to produce ultraporous implants, theoretically increasing the rate of bony ingrowth and emulating the elastic modulus of the bone. However, the increased porosity may weaken the stem structure, predisposing to fracture and implant breakage.

Investigations evaluating the outcomes of these powder-made stems are still lacking in literature. In particular, no in vivo assessment about survival rates and the reasons for revision of EBM stems was performed. Thus, a prosthetic registry was queried about powder-technology-built stems, in order to investigate the rate of mid-term complications, aseptic stem loosening, and implant breakage, using conventional stems as a control group.

## Material and methods

The Registry of Prosthetic Orthopedic Implants of Emilia Romagna (RIPO) was queried about all cementless stems implanted in Emilia Romagna from 1 January 2010 to 31 December 2017 only for Emilia Romagna residents to control out-of-region patients’ follow-up loss [11]. All patients surgically treated during this period were included without applying minimum follow-up. Titanium alloy stems fabricated using EBM technology (Parva, Pulchra) were evaluated and compared with all cementless stems implanted during the same period (control group) (Table 1). RIPO has been collecting all prosthetic primary and revision implants in the Emilia Romagna region (including 68 orthopedic departments) since January 2000. It is a member of the International Society of Arthroplasty Registries [12]; and collaborates on the evaluation and risk prevention of the main prosthetic implants [13–15]. The register records not only the prosthetic implant features, but also the clinical patient features, the surgical procedure, and the type of fixation, with a capture rate of 98% [16].

**Table 1:** Population under study

	Groups	
	Standard cementless stems	EBM stems
Implants *n*° (%)	37768 (98.4)	597 (1.6)
Average age (years) (min–max)	68.7 (13–99)	60.6 (19–87)
Sex		
Female (%)	58.1	47.3
BMI		
Overweight and obese (%)	62.6	61.9
Head size~		
≥36 (%)	52.1	65.2
Weight^		
≥80 kg (%)	38.0	41.5

Descriptive statistics

### EBM-built stem features

**Fig. 1:**
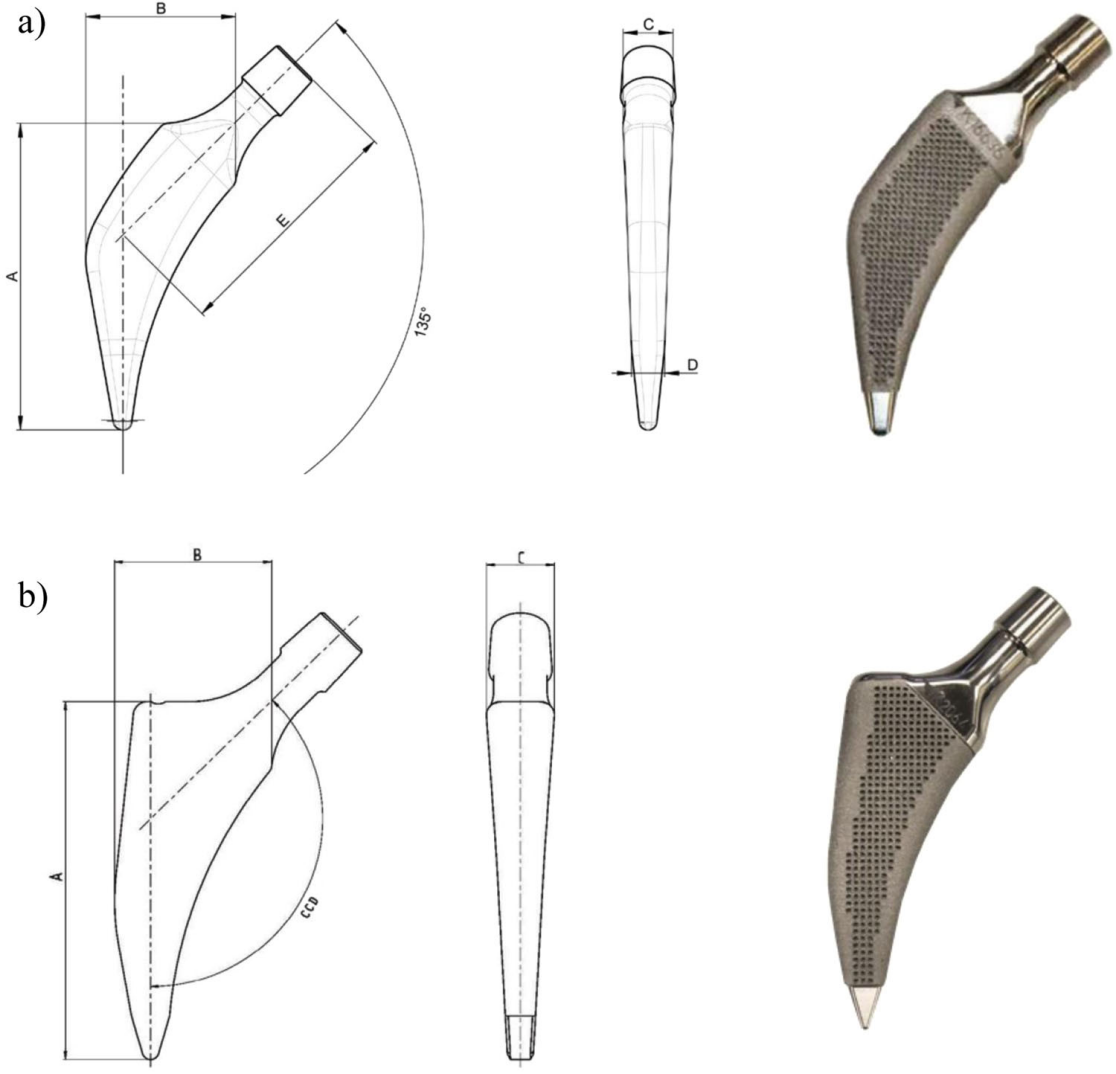
Design and structure of the two powder-made stems. The two powder-made stems have a similar three-dimensional structure with an ultraporous surface, but the design is different: **a** Parva, Adler Ortho, Milan Italy; **b** Pulchra, Adler Ortho, Milan Italy

The two stems under examination are both EBM-built short stems: one short bone-conserving stem (neck-preserving stem: Parva, Adler Ortho, Milan Italy) and one short stem with standard resection (Pulchra, Adler Ortho, Milan Italy) (Fig. 1). The bone-conserving stem (Parva) is available in 11 different sizes from 8 to 18. Each size has two different configurations, depending on the offset: short and standard. The standard short stem (Pulchra) is a calcar loading stem available in 12 sizes. Standard and offset versions are available for each size. Both implants use EBM technology and the material surface has the same characteristics. The mechanical characteristics of the stems are summarized in Table 2.

**Table 2:** EBM stems features

Fixation	Press-fit
Material	Ti6A14V
Porosity	65%
Pore size	700 µm
Tensile stress resistance (according to ASTM F1147-05)	64.2 Mpa
Shear stress resistance (according to ASTM F1144-05)	39 MPa
Shear stress fatigue resistance (according to ASTM F1160-05)	No damage
Abrasion resistance (according to ASTM F1978-00)	17.1 mg

Both stems passed the conformity tests to obtain the CE mark (ISO 7206-4 ed ISO 7206-6)

### Statistics

Demographics, implant features, and reasons for revision were recorded. The survival analysis was performed using the Kaplan–Meier method. The primary endpoint was stem revision, with a focus on breakage and aseptic loosening. Ninety-five-percent confidence interval was established for all required distributions. Revision of the cup/insert was not considered as failure. Implants were followed until the last date of observation (date of death or 31st December 2017). Statistical analyses were performed using SPSS 14.0, version 14.0.1 (SPSS Inc, Chicago, IL) and JMP, version 12.0.1 (SAS Institute Inc, Cary, NC, 1989–2007). Wilcoxon tests assessed the statistically significant differences between the survival curves: the significance threshold (*p*) was set at 0.05.

Approval of the institutional review board was not necessary, as RIPO respects the standard levels of ethics and conceals patients’ identity as a standard practice.

## Results

A total of 38,365 cementless stems implanted between 1 January 2010 and 31 December 2017 were evaluated. Five hundred and ninety-seven stems were made with EBM technology (Parva and Pulchra), standard cementless stems were 37,768.

**Fig. 2:**
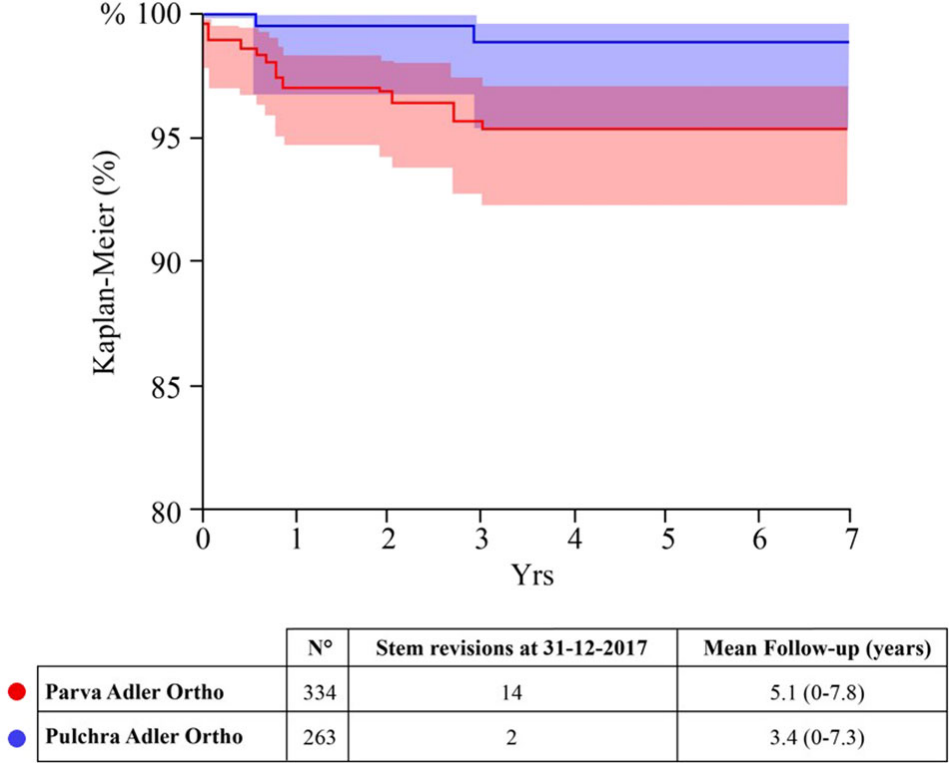
Survival comparison between the two powder-made stems over time. There were more Parva stems failures even though the difference is not statistically significant between the two populations

The mean follow-up of EBM-built stems was 4.3 years (range: 0–7.8) (Table 3). Out of 597 additive manufacturing stems, 16 revisions were performed (2.7%). Fourteen revisions involved the neck-sparing stem, only two reimplantations occurred in the standard resection short stem cohort (Fig. 2). The main cause of revision for powder-made stems was aseptic loosening of the stem (62.5%, 1.7% of the all primary implants). No mechanical failures were recorded in the EBM cohort.

**Table 3:** Cohorts, revision rate, and mean follow-up

	*N*°	N. of stem revisions at 31 December 2017	Mean follow-up and range (years) of implants in full cohort
Standard cementless stems	37,768	627	3.6 (0–8.0)
Powder-made stems	597	16	4.3 (0–7.8)

The mean follow-up of standard cementless stems was 3.6 years (range: 0–8.0). Out of 37,768 cementless stems implanted, 627 revisions have been performed (1.7%). The three main causes of revision were aseptic loosening (0.5%), periprosthetic bone fracture (0.3%), and prosthesis dislocation (0.2%).

**Fig. 3:**
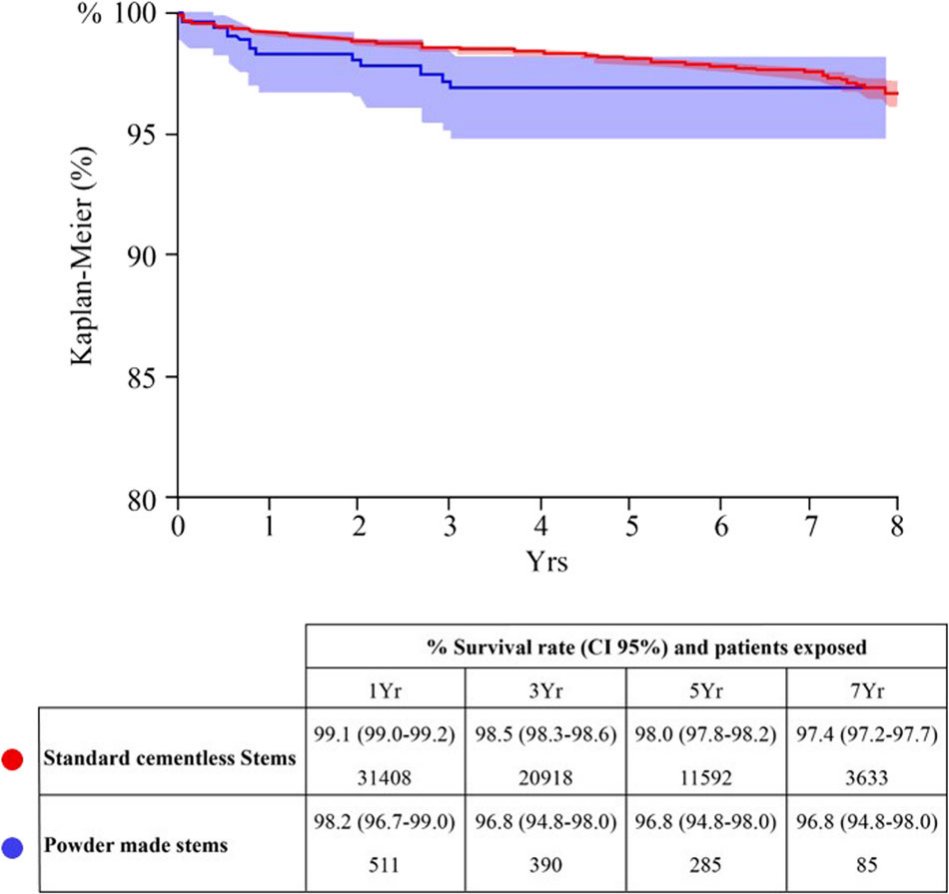
Global survival rate. The graph compares the two populations under study and evaluates their survival rate over time

EBM-built stems did not show a statistically significant difference in overall survival rate (*p* > 0.05) when compared to the other cementless stems at 5 years follow-up: hazard ratio (age- and gender-adjusted): 1.3 (0.8–2.1); *p* = 0.2642 (Fig. 3).

## Discussion

EBM technology was introduced to achieve maximum porosity with minimum usable surface, reducing material waste and expense [17]. As highlighted by Castagnini et al., EBM technology allows for larger porosity, more uniform and bone-like elastic modulus but, on the other side, may be more prone to delamination and cracking [18]. Taniguchi et al. [7] in their in vivo study and Frosch et al. in an vitro study [8] demonstrated that porous titanium implants with a pore size of 600 µm (P600) showed significantly higher fixation and bony ingrowth than those with different pore sizes. These outcomes were also confirmed when EBM-built cups were evaluated: a lower rate of aseptic loosening was recorded at 7-year follow-up in a registry study, and dependable signs of osseointegration were evident at a minimum follow-up of 3 years [9, 10]. Thus EBM could create a new bulk construct by a range of porosity and pore size, impacting the bony fixation and reducing the stiffness of the metal implants [19, 20]. However, some concerns about mechanical strength still exist, in particular involving the stems and the breakage risk of the implants due to delamination and crack propagation, which are the possible negative side of ultraporous devices produced using additive manufacturing. To date, studies including large cohort of EBM-built femoral implants are still lacking and no clinical data about EBM stem mechanical failures on large numbers are available.

We wondered if the titanium alloy stems produced using additive technology (EBM) still achieved low rate of prosthetic aseptic loosening and a negligible occurrence of implant breakage. In order to provide a reliable comparison, a control group of cementless femoral implants was selected.

The rate of aseptic loosening was not statistically different in the two cohorts. Most of all, no events related to mechanical failure occurred in the EBM cohort [21]. This is a very important finding, as EBM-made device is theoretically more prone to fatigue failure due to crack propagation, as a consequence of manufacturing technique.

**Fig. 4:**
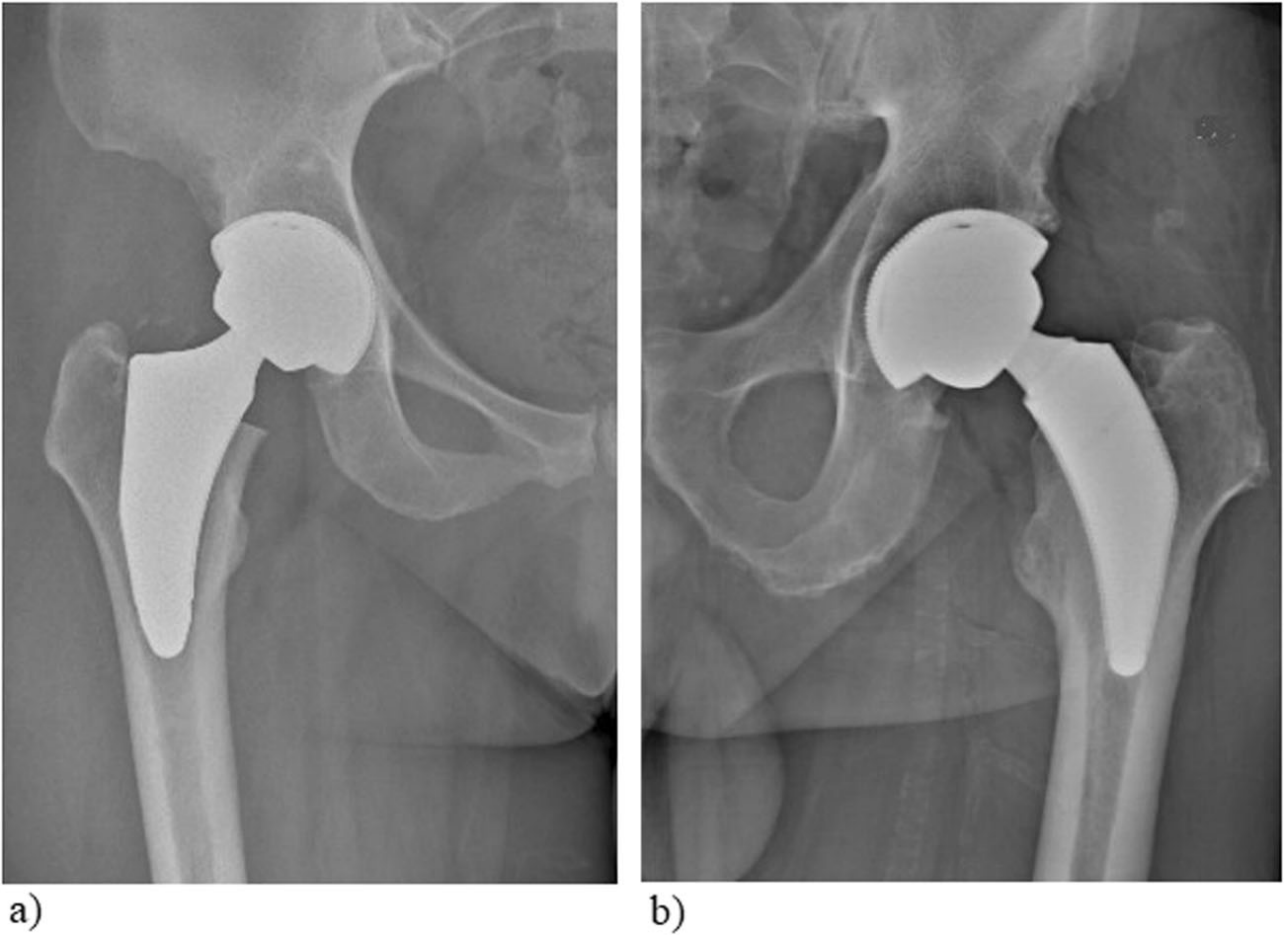
Radiographic images of the stems 2 years after surgery. **a** Pulchra, Adler Ortho, Milan Italy, **b** Parva, Adler Ortho, Milan Italy

Moreover, the dependable outcomes highlighted by the present registry report involving EBM short stems were comparable with those found in literature regarding the revision rates of short stems not produced using additive manufacturing and EBM technology, reporting a 5-year survival rate of 96.8% (CI) (Fig. 4) [22].

In general, short and ultra-short stems have showed positive results in many large cohorts. In a registry study involving 57,359 implants, comparing conventional stems and short stems, Giardina et al. [16] showed that survival rates were similar in all the cohorts analyzed. In their series of 261 patients with short stem implants (Tri-Lock BPM DePuy^®^), Amendola et al. [23] experienced an excellent implant fixation but observed a 9% rate of thigh pain.

It should be clearly stated that one of the two EBM-built stems under study (the short bone-conserving stem, Parva) showed a revision rate of 4.2% at a mean follow-up of 5.1 years, much higher than the other short stem involved. We suppose that the outcomes of this neck-sparing stem were mainly due to the demanding surgical technique. As observed by Huo et al. [24] neck-sparing stems have a greater risk of undersizing as the surgeon settles for minimum stability and avoids further broaching, fearing lateral cortical fracture. Thus, the risk of malseating, inaccurate position, and undersizing may lead to more revisions, in particular due to aseptic loosening, even at short term. Technical issues affecting the higher rates of aseptic loosening in short stems were suspected also in a meta-analysis by Khanuja et al. [25]. As conflicting data on short bone-conserving stems were reported (a short-term survival rate: 83–100%, depending on the prosthetic design), Khanuja et al. concluded that short bone-sparing stems require greater technical expertise and are less forgiving than standard stems due to implant misalignment, incorrect sizing, neck encumbrance. However, this speculation cannot be confirmed by the present registry study and requires radiographic assessments.

This report is the first study providing the mid-term outcomes of two titanium alloy EBM-built stems on a large population of patients. These stems show a reassuring safety profile, with no increased risk of aseptic loosening and no mechanical failures recorded at a mid-term follow-up.

However, the study presents some notable limitations related to the nature of registry study. The most important limitation is related to the lack of clinical and radiographic data, which could not be provided due to the registry nature of the study. Thus, no evidence about thigh pain and bone remodeling around the stems is available: these two aspects of the EBM stems should be investigated in appropriate clinical trials. Moreover, the different follow-ups of the two cohorts prevent from drawing a precise and definitive comparison between EBM and conventional stems. The mid-term follow-up due to the recent introduction of this technology did not allow the evaluation of any late complications. However, the patients at risk in the EBM cohort are still numerous at a 7-year follow-up (85), providing a quite reliable assessment at a mid-term perspective [21].

In summary, the EBM technology, which was previously described for acetabular sockets, provided dependable outcomes even for femoral stems at midterms. The rate of aseptic loosening of the powder-built stems was not inferior to conventional femoral devices. Most of all, no mechanical failures were recorded at midterms. So, even if new studies will be needed extending the follow-up and integrating clinical and radiographic outcomes, powder-built stems appeared to be safe devices at midterms.
